# CRISPR/Cas9 mediated generation of an ovine model for infantile neuronal ceroid lipofuscinosis (CLN1 disease)

**DOI:** 10.1038/s41598-019-45859-9

**Published:** 2019-07-09

**Authors:** S. L. Eaton, C. Proudfoot, S. G. Lillico, P. Skehel, R. A. Kline, K. Hamer, N. M. Rzechorzek, E. Clutton, R. Gregson, T. King, C. A. O’Neill, J. D. Cooper, G. Thompson, C. B. Whitelaw, T. M. Wishart

**Affiliations:** 1 The Roslin Institute and Royal (Dick) School of Veterinary Studies, University of Edinburgh, Edinburgh, UK; 2 Centre for Discovery Brain Science, University of Edinburgh, Hugh Robson Building, Edinburgh, UK; 3 Euan MacDonald Centre for Motor Neurone Disease Research, University of Edinburgh, Edinburgh, UK; 4 Centre for Clinical Brain Sciences, University of Edinburgh, Chancellor’s Building, 49 Little France Crescent, Edinburgh, UK; 5 Royal (Dick) School of Veterinary Studies, The University of Edinburgh, Edinburgh, UK; 6 Wellcome Trust Critical Care Laboratory for Large Animals, Roslin Institute, Easter Bush, Edinburgh, UK; 7 BioMarin Pharmaceutical Inc, San Rafael, CA USA; 8 Los Angeles Biomedical Research Institute at Harbor-UCLA Medical Center, David Geffen School of Medicine, UCLA, Torrance, CA USA; 9 Department of Pediatrics, Washington University School of Medicine, St Louis, MO USA

**Keywords:** Neurodegeneration, Genetic predisposition to disease

## Abstract

The neuronal ceroid lipofuscinoses (NCLs) are a group of devastating monogenetic lysosomal disorders that affect children and young adults with no cure or effective treatment currently available. One of the more severe infantile forms of the disease (INCL or CLN1 disease) is due to mutations in the palmitoyl-protein thioesterase 1 (*PPT1*) gene and severely reduces the child’s lifespan to approximately 9 years of age. In order to better translate the human condition than is possible in mice, we sought to produce a large animal model employing CRISPR/Cas9 gene editing technology. Three *PPT1* homozygote sheep were generated by insertion of a disease-causing *PPT1* (R151X) human mutation into the orthologous sheep locus. This resulted in a morphological, anatomical and biochemical disease phenotype that closely resembles the human condition. The homozygous sheep were found to have significantly reduced PPT1 enzyme activity and accumulate autofluorescent storage material, as is observed in CLN1 patients. Clinical signs included pronounced behavioral deficits as well as motor deficits and complete loss of vision, with a reduced lifespan of 17 ± 1 months at a humanely defined terminal endpoint. Magnetic resonance imaging (MRI) confirmed a significant decrease in motor cortical volume as well as increased ventricular volume corresponding with observed brain atrophy and a profound reduction in brain mass of 30% at necropsy, similar to alterations observed in human patients. In summary, we have generated the first CRISPR/Cas9 gene edited NCL model. This novel sheep model of CLN1 disease develops biochemical, gross morphological and *in vivo* brain alterations confirming the efficacy of the targeted modification and potential relevance to the human condition.

## Introduction

The neuronal ceroid lipofuscinoses (NCL) are a group of predominantly autosomal-recessive storage disorders which mainly affect children and young adults, and are notably the most common form of childhood dementia^1^. Children who suffer from this neurodegenerative condition experience psychological and motor deficits, loss of vision and seizures leading to a profound impact upon the CNS and ultimately a premature death. The age of onset and rate of disease progression are governed by which ‘CLN’ gene is deficient and the precise mutation that is present. Mutations in the *CLN1/PPT1* gene are responsible for one of the most severe and early onset forms of the disease, with clinical onset between 12–24 months of age^2^. These children have a severely reduced lifespan, with death occurring at approximately 9 years of age^3^. Currently there is no cure for the disease.

Diagnosis of INCL can be elusive, however there is a reliable blood test available that can be employed for pre and post-natal diagnosis^4^. This assay detects a significant reduction in the activity of the enzyme palmitoyl-protein thioesterase 1 (PPT1) in leucocytes from CLN1 patients, which is a direct consequence of mutations in the *PPT1* gene^5^. Many other disease-specific hallmarks exist; some are detectable with specialist imaging techniques whilst others are more readily seen at autopsy. These include, intracellular accumulation of autofluorescent storage material and regional neuroanatomical alterations^6^.

In order to generate a model of the human condition and enable pre-symptomatic investigations for evaluation of potential therapeutic interventions^7^, a *PPT1* null mutant mouse model was produced^8^. These mice have been valuable for defining progressive neuropathological and behavioral changes, and for testing experimental therapies^2^. However, the extent of neurodegeneration in these mice, even at disease end stage^9,10^, does not reach the full devastating extent seen in human INCL. Larger animal species have advantages over mice as disease models, and two naturally occurring cases of CLN1 disease have also been reported in dogs - a male daschund with a unique mutation not previously reported in humans and one female cane corso with an alteration detected in 2 human cases^11,12^ (NCL Mutation Database, http://www.ucl.ac.uk/ncl/). However, as far as we are aware, neither of these unique animals are currently available as models that can be studied. Naturally occurring ovine models of CLN5 and CLN6 forms of NCLs do exist and have proved valuable for studying the pathogenesis of these disorders and the pre-clinical development of therapies including viral mediated gene transfer^13–16^.

In this study we have attempted to reproduce the complex nature of the disease seen in INCL patients by generating a large animal model that carries disease-causing mutations present in human CLN1 disease. We describe how the CRISPR/Cas9 system was used to introduce the commonly occurring human R151X mutation into the ovine PPT1 locus^5,17^. We then demonstrate that the downstream biochemical and regional morphological brain alterations commonly reported in human patients are present in this novel ovine model at a humanely defined disease endpoint. This is the first time that the CRISPR/Cas9 system has been used to generate a model of CLN1 disease or similar lysosomal storage disorders. These novel CLN1 sheep will not only help further our understanding of INCL pathogenesis, but also have the potential to enable therapeutic development for this devastating pediatric disorder.

## Results

### Generation of PPT1 sheep

**Figure 1:**
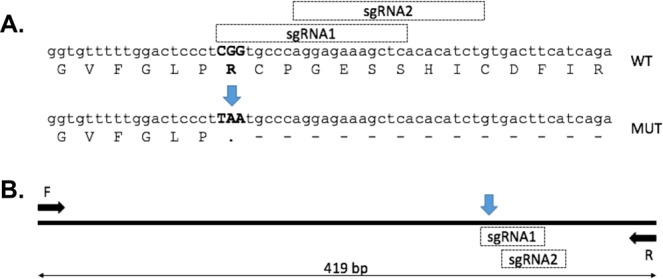
CRISPR/Cas9 engineering of PPT1 sheep (**A)** sgRNAs were designed proximal to the PPT1 target site on *Ovis aries* chromosome 1 (indicated by blue arrow). (**B)** Sheep zygotes were injected with Cas9 mRNA, sgRNA1 or sgRNA2 and an HDR (homology directed repair) template (90mer ssODN).

As homology DNA repair (HDR) is believed to be most efficient immediately proximal to a double strand DNA break (DSB), two alternative single stranded guide RNAs (sgRNAs) were designed as close as possible to the coding sequence for R151 of sheep PPT1 (Fig. 1A). The CCTop CRISPR/Cas9 online tool (https://crispr.cos.uni-heidelberg.de/) predicts a low probability of off-target cutting by either of these guides within exonic sequence of the sheep genome. CRISPR reagents (sgRNA (MEGAshortscript T7 transcription kit, Life Tech.) + ssODN (single strand Oligodioxynucleotide; IDT) + Cas9 mRNA (CRISPR Associated protein 9 mRNA; PNA Biosciences)) were microinjected into the cytoplasm of *in vitro*-derived sheep zygotes as previously described^18^, with no evidence of toxicity observed during subsequent culture. Sixty-six blastocysts were transferred to 33 hormonally synchronized recipient ewes^18^, with 19 (58%) carrying a pregnancy to term. Twenty-four lambs were genotyped by PCR of genomic DNA and sequencing (Fig. 1B), revealing 12 (50%) that were not edited, 3 (12.5%) had random insertions or deletions (indels) at the target locus, 6 (25%) had undergone HDR to introduce the stop codon at the target locus of a single allele and 3 (12.5%) had undergone HDR on both alleles (Table 1). All lambs were grossly phenotypically normal at birth.

**Table 1:** Editing the sheep PPT1 locus.

	Blastocysts	Recipients	Pregnancies	Lambs	Genotypes			
					WT	Indel	Het HDR	HOM HDR
sgRNA1	35	17	10	10	3	3	2	2
sgRNA2	31	16	19	14	9	0	4	1

Details of blastocysts, recipients, pregnancies and the geneotypes of liveborn offspring for each sgRNA. WT: wild type; Indel: insertion and/or deletion; Het HDR: heterozygous homology dependant repair; Hom HDR: homozygous homology dependent repair.

### Confirmation of CLN1 phenotype

**Figure 2:**
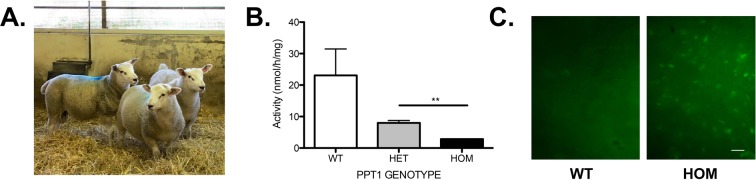
Confirmation of CLN1 disease. (**A)** Asymptomatic homozygote PPT1 sheep. (**B**) PPT1 enzyme activity assay tested in plasma (20ug protein) detected significant differences between heterozygote and homozygote sheep (P = 0.0025). Statistical analyses utilized unpaired two-tailed Student’s t-test. Error bars represent the standard error of the mean (SEM). (**C**) Autofluoroscent inclusion bodies are detected in homozygote sheep scale bar represents 50 microns.

A one step fluorometric PPT1 enzyme activity assay^4^ was employed in wild type, heterozygote and asymptomatic homozygote PPT1 sheep plasma at approximately 1 year of age (Fig. 2). There was a significant decrease in PPT1 enzyme activity in homozygous vs. heterozygote sheep (P = 0.0025) (Fig. 2), consistent with the near complete reduction in activity observed using this assay in the human patient population^4^. Another characteristic hallmark of CLN1 disease is the accumulation of autofluorescent storage material within lysosomes. This was also detected in the spinal cords of the homozygote sheep (Fig. 2).

Visual function was assessed via remote observation of navigation and visual tracking alongside menace response testing, whilst pupillary light and dazzle reflex testing was used to assess subcortical responses to light and glare, respectively^16,19,20^. By 12 ± 1 months of age, homozygote sheep were observed bumping into obstacles followed by a significant deficit in the menace response by 17 ± 1 months at the humanely defined endpoint (Supplementary Fig. 2). Motor performance and vestibular system integrity were assessed remotely by observation of gait and balance, followed by direct testing of conscious proprioception using hoof placement, knuckling and hopping tests, and balance via a ‘push test’^20^. There was a significant increase in proprioceptive deficit in the homozygote sheep (Supplementary Fig. 2).

### Regional specific disease alterations are observed in PPT1 sheep brains

**Figure 3:**
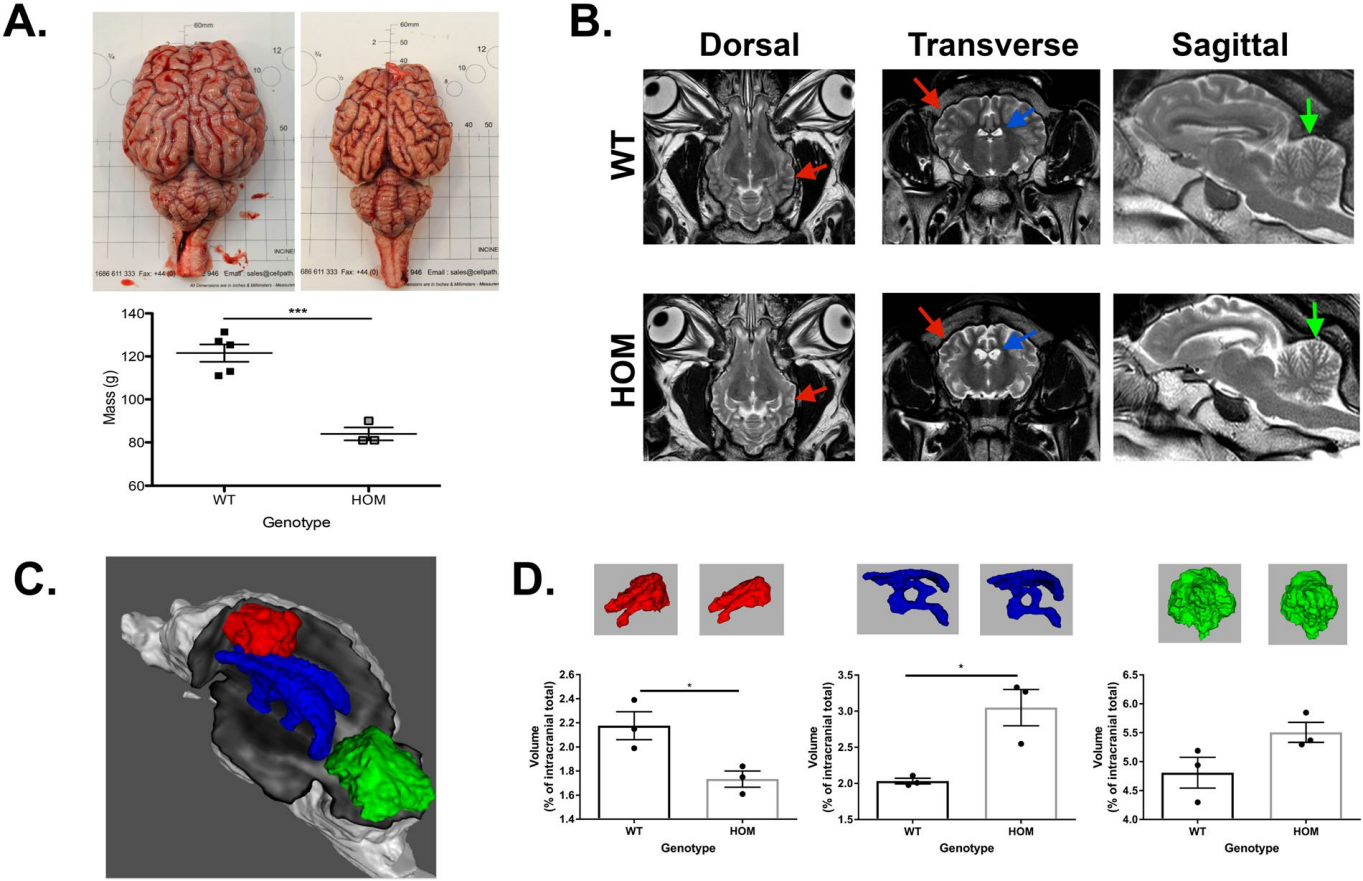
Selective pattern of neurodegeneration is observed in ovine model of INCL. (**A**) Gross anatomical disparity identified between WT control (left) and PPT1 homozygote (right) brains. Total fresh brain weight is significantly reduced in homozygote sheep shown in scatter plot (P = 0.0006). (**B**) Comparison of orthogonal T2-weighted spin echo imaging slices in example wild type (top row) and homozygous (bottom row) animals. There is global reduction in cerebral volume (red arrows), with ventriculomegaly (blue arrows) and thinning of the frontal cortex in the homozygote sheep. Note increased fluid signal between cerebellar folia (green arrows) suggestive of neocerebellar atrophy. (**C**) Volumetric renderings showing regions of interest for bilateral motor cortex (red), cerebellum (green), and tri-ventricular cerebrospinal fluid (blue). (**D**) Scatter plot with bar chart of volumetric data for the bilateral motor cortex, triventricular CSF and cerebellum with corresponding 3D images above each data point relating to the area and genotype. Statistical analyses utilized unpaired two-tailed Student’s t-test. Error bars represent SEM.

The homozygote sheep and WT controls were euthanised at 17 ± 1 months in accordance with home office specification when a moderate phenotype was observed. Gross morphological assessment of the homozygote resected brains at *post mortem* showed a statistically significant 30% decrease in mass compared with age-matched wild type controls with pronounced regional atrophy of the neocortex (Fig. 3A). Qualitative imaging assessment by MRI identified a global reduction in brain volumes, with enlarged ventricles, and thinned grey matter, particularly in sensory regions of the neocortex caudal to the central sulcus (Fig. 3B). The hindbrain, by comparison was relatively normal although there appeared to be some atrophy of the neocerebellum (Fig. 3B).

Quantitative Imaging analysis using MATLAB software allowed more detailed volumetric evaluation of specific regions of interest: the motor cortices, triventricular spaces and cerebellum (Fig. 3C). The volumetric analyses of the homozygous sheep demonstrated significantly lower cerebral volume with an average 20% decrease at the time of imaging (Fig. 3D). The triventricular cerebral spinal fluid (CSF) space in the homozygotes was significantly increased on average by 50%, however these sheep had not developed clear differences in overall cerebellar volumes at this time point (Fig. 3D). Features in this model recapitulate the gross anatomical findings in a naturally occurring ovine model caused by mutation of the CLN6 gene^21^.

## Discussion and Conclusions

Here we have shown for the first time that CRISPR/Cas 9 engineering can effectively produce a large mammalian model of childhood dementia by introducing a disease-causing mutation present in human cases. This new ovine model shows a regionally-selective pattern of neurodegeneration, and relevant clinical signs similar to those reported in human patients with INCL^2^. Taken together our data suggest a more robust recapitulation of human disease than is shown by the existing CLN1 mouse models. Certainly, even without detailed neuropathological assessment, the degree of cortical atrophy is much more pronounced than in PPT1 deficient mice^9,10^, perhaps due to disparities in rodent brain anatomy when compared to humans^22^. These CLN1 data resemble the much more pronounced extent of cortical pathology in CLN6 deficient sheep compared to *nclf* mice^23,24^.

By using CRISPR/Cas9 editing we have successfully incorporated the most common human disease-causing mutation in the gene, *PPT1*, resulting in development of clinical signs and a humanely defined endpoint of 17 ± 1 months, approximately 10% of the lifespan of a wild type control sheep. This is comparable to the reduced lifespan of human INCL patients who on average die at 9 years of age, approximately 11% of the average human life expectancy^25^. By comparison the existing murine model reaches a terminal stage by 8 months which is approximately 33% of its potential life span^8^. Other principal phenotypic characteristics of the human disorder observed in this PPT1 sheep model include accumulations of autofluorescent material, visual impairment and brain region-specific volume alterations^3^. These CLN1 sheep have an overall difference in brain mass of 30% at necropsy. This again more closely resembles the severity of human disease^2^, compared to the reductions in brain mass observed in the murine model^9^.

MRI has been informative in following disease progression in INCL patients^26^. Our imaging data mirror those seen in a related naturally occurring CLN5 ovine model, and the dramatic impact of CLN1 disease upon the volume of the cerebral cortex^27–29^ with more subtle effects upon the cerebellum. The onset and progression of such regional effects upon the CLN1 sheep brain will be important to define, both via imaging and pathological studies, and these data will be very informative for the targeting of experimental therapies.

Many of the difficulties associated with neurodegenerative disease research (at the level of understanding disease progression and subsequent intervention development) have been attributed to the use of models that do not faithfully recapitulate the human condition^30^. Diseases that naturally occur in large animals are extremely useful for determining pathogenic events and drug discovery driven research^16,31^, and the pre-clinical work done in CLN2 deficient dachshunds prior to FDA approval of ERT exemplifies this^31,32^. However, with the advent of gene editing technologies we are no longer limited to identifying naturally occurring forms of genetic disease, which may not bear the same mutations seen in the patient population^15^. Thus, models can now be generated with increasing genetic specificity by recreating human disease-causing mutations^33^ as we have done for the first time in CLN1 disease sheep.

Such methods have already proven to be more effective in the development of two GM porcine models; Huntington disease and Ataxia telangiectasia (AT)^34,35^. These large animal models display disease phenotypes inadequately represented in commonly used murine systems for the same conditions. These include deficits in cerebellar cytoarchitecture detected at birth in the AT model, and a respiratory difficulty phenotype which is a secondary characteristic of human Huntington disease. Thus, although the financial burden of generating models in larger animal species is initially high, these models have considerable potential to substantially reduce the overall socioeconomic burden of such neurodegenerative disorders.

Here we have shown that CLN1 disease sheep, created using CRISPR/cas9 technology to bear a mutation causing a human neurodegenerative disorder, recapitulates disease-specific hallmarks including clinical signs and morphological changes as determined by neuroimaging and gross pathology. Whilst our data from end-stage sheep are encouraging, future work including in depth neuropathological, molecular and MRI studies throughout the lifespan of affected animals will more fully define the validity of this novel model. These additional studies should permit earlier detection of divergence from normal brain development, and potentially yield more sensitive and specific biomarkers for disease progression and landmarks to judge efficacy in subsequent preclinical therapeutic trials.

## Methods

### CRISPR/Cas9 production of PPT1 sheep

Two sgRNAs were designed and synthesised in order to target SpCas9 to the coding sequence for PPT1 R151 on chromosome 1 of the *Ovis aries* genome (sgRNA1 gagctttctcctgggca and sgRNA2 agatgtgtgagctttctcct). A specific ssODN was designed for each of the two alternative guides (Supplementary Fig. 1), and sgRNA + ssODN + Cas9 mRNA were co-injected into the cytoplasm of sheep zygotes. At day 6–7 post injection blastocysts were surgically transferred to synchronous ewes as previously described^18^, resulting in 19 pregnancies. Genomic DNA was prepared from ear notches of each of 24 lambs. Genotyping was by PCR spanning the target site with primers F (ccaagacccaagaggtgaga) and R (gtgacagttccgcatccttt) to give a 419 bp fragment that was then sequenced.

### Analysis of autofluorescent storage material

Cervical, thoracic and lumbar portions of spinal cord were excised *post mortem* and fixed in formal saline for 10 days. Thereafter, tissues were transferred to a 50 mM tris, 0.05% azide buffer (pH7.6) for long term storage at 4 °C. Cervical spinal cord from homozygote and age- matched controls were trimmed sagittally into approximately 1 mm thick sections and mounted onto glass slides with vectashield fluorescent mounting medium (Dako). Autofluorescent storage material was visualized using a fluorescent microscope at X10 magnification (Leica DMLB microscope with Hammamatsu camera ORCA-ER attachment). Images were captured using Image J and Micromanager 1.4.18 software with a lumencore spectraX light source and GFP filter at excitation 485 nm and emission 520 nm.

### PPT1 enzyme assay

Blood was collected by venipuncture. Heparin-treated blood was centrifuged in Vacutainers at 1000 × *g* for 15 min at 4 °C to isolate the plasma. Protein determination was carried on the plasma using micro BCA assay (Life technologies, UK). The 1 step PPT1 assay protocol was employed as described in^4^. Briefly, 20ug of plasma was added to 20ul of substrate solution (0.64 mM MU-6S-Palm ß Glc, 15 mM dithiothreitol (DTT), 0.375% (w/v) Triton X-100, and 0.1 ß glucosidase from almonds (Sigma) in McIlvain’s phosphate/citrate buffer (pH 4.0). The reaction was incubated overnight (18–24 hours) at 37 °C and terminated by addition of 0.5 M NaCO_3_/NaHCO_3_ (pH 10.7) containing 0.025% TritonX-100. Samples were read on a fluorometer at 405 nm (360 nm excitation and 460 nm emission).

### Magnetic resonance imaging (MRI)

#### Imaging subjects

Six animals (3 wild type and 3 homozygous mutants, M:F 2:1) were imaged at approximately 480 days. This time point was selected to provide the most animals in a specific 2 week period. In addition, this time point was similar to that used in another ovine study of naturally occurring CLN6 model (16 month)^21^.

#### Anaesthesia

The sheep was sedated with medetomidine (5µgkg-1; “Medetor”; Chanelle, Berkshire), ketamine (1mgkg-1; “Ketamidor”; Chanelle, Berkshire) and midazolam (0.25^mgkg-1^; “Hypnovel”, Roche, UK) which were combined and administered slowly “off the needle” into the jugular vein. Sternal recumbency developed over 5 minutes. A jugular cannula was placed and anaesthesia induced with 3^mgkg-1^ IV propofol (“Propoven 1%”; Fresenius Kabi, Cheshire). Once unconscious, the trachea was intubated with an 8 mm endotracheal tube (ETT) and the cuff inflated. Anaesthesia was initially maintained using isoflurane (“IsoFlo”; Zoetis, Surrey) vaporized in oxygen (FiO_2_ > 0.95) administered via a Bain breathing system; the animals breathed spontaneously. Once in the scanner, the ETT was connected to a circle breathing system, and anaesthesia maintained with 1.5–2% isoflurane vaporized in 2 L/min oxygen. Mechanical ventilation was imposed using an MRI-compatible ventilator (Penlon 200) to maintain normocapnia. Fractional concentrations of end-tidal carbon dioxide (Fe’CO_2_) were monitored continuously throughout scanning (lasting 40 minutes) but other physiological variables were assessed intermittently because access was limited.

After scanning, isoflurane administration was ended, the sheep was removed from the scanner and positioned in sternal recumbency before IV atipamazole (25^µgkg-1^; “Antisedan”, Vetoquinol, Buckinghamshire) was administered. The animals continued to breathe 100% oxygen until active chewing/swallowing prompted tracheal extubation. The ETT was withdrawn with the cuff partially inflated in order to remove accumulated oropharyngeo-trachaeal fluid.

#### Image acquisition

MRI were acquired on a 1.5 T Philips Achieva (Philips, Best, The Netherlands) using a FlexS surface coil. Transverse T2-weighted (T2w) fast spin echo (FSE) images (TR = 5421 ms, TE = 100 ms, NSA = 4, slice thickness 3.85 mm, voxel size 0.41 × 0.41 mm, field of view 150 × 150 × 100 mm) were acquired, in addition to sagittal and dorsal imaging with comparable weighting and imaging parameters. Transverse FLAIR (fluid attenuated inversion recovery), and T1-weighted fast spin echo images were acquired before and after intravenous administration of gadopentetate dimeglumine (0.5 mmol/mL; “Magnevist”, Bayer, Berkshire).

#### Image analysis

Qualitative assessment was performed by an expert neuroradiologist with 11 years’ experience (GT). For quantitative volumetric analysis, an in-house pipeline was developed. Using SPM12 (Statistical Parametric Mapping, The Wellcome Trust Centre for Neuroimaging, University College London, UK) with Matlab 2017a (MathWorks, Natick, MA, USA,) the transverse T2w images were up-sampled using linear interpolation to high resolution isotropy, matching the through plane to the in-plane voxel size. The dorsal and sagittal images were linearly co-registered to this with 6 degrees of freedom using SPM12 to produce spatially-congruent, uniform, high resolution triplanar orthogonal images^36–38^. Matlab was used to estimate an isotropic image from these using wavelet-based fusion reconstructions^39^. Expert manual intracranial segmentations were performed on all three orthogonal planes of the T2w SE imaging, and the SPM (Statistical parametric mapping) linear transforms were used to produce a high-resolution whole brain mask in isotropic space, binarized by thresholding at 0.5. In the absence of a bespoke template and atlas for the specific breed used here the University of Melbourne Corriedale ovine atlas from Liyanage *et al*. was chosen, since this contained regions of interest (ROI) relevant to the expected areas of pathological brain involvement^40^. The Melbourne template does not include a T2w image, however, and a template including a brain-extracted T2w from INRA (Institut National de Recherche Agronomique, France) was chosen to improve accuracy of template-based segmentation^41^. The Melbourne template was warped into INRA T1w template space using ANTs (Advanced Normalization Tools, Penn Image Computing & Science Lab, University of Pennsylvania, USA) diffeomorphic registration with affine and SyN algorithms, and cross correlation similarity metric^40–43^. The resultant series of transforms produced the Melbourne atlas regions of interest in INRA template space. Individual wavelet-reconstructed T2w high resolution isotropic images for each sheep were non-linearly warped into INRA T2w template space using ANTs. The inverse concatenation of the resulting linear transforms and nonlinear warps were used to produce native space Melbourne atlas segmentations for each subject using nearest neighbor interpolation. Each segmentation was individually reviewed for artefact and accuracy. Based on qualitative assessment, and given the sample size, comparisons were made between supraventricular cortex (bilateral motor cortex), tri-ventricular CSF, and the cerebellum atlas regions. Matlab was used to calculate ROI (Region of Interest) volumes for comparison. Each animal’s regional volumetric data was normalised to their individual intracranial volume and converted to a percentage to reduce variability^44^. With two male and one female animal in each group, the effect of sex was not examined; in prior work with a related naturally-occurring ovine model, sexual dimorphism was shown to contribute less to brain volume variance than that expected from pathological differences in affected sheep^21^.

#### Brain atrophy analysis

Brains were excised at post mortem with the pituitary gland detached (Fig. 3). Each brain was weighed and a photograph taken on Dispocut white boards (CellPath, UK). Statistical tests were performed in GraphPad Prism software. For all analyses P < 0.005 were considered statistically significant.

#### Clinical observations

Weekly clinical assessment commenced once a phenotype was observed in the first homozygote sheep and was performed by veterinary clinicians including a certified or board-eligible veterinary neurologist, together with a board-eligible small ruminant specialist. Assessments included hands-off observation of mentation, behaviour, gait, balance, and navigation, followed by a general physical examination and full neurological examination as described by Crilly *et al*.^20^ (See Supplementary Fig. 2). In addition, objective scores of clinical abnormality (0–4; where 0 = normal and 4 = severe) were obtained for (i) deficits in vision and and subcortical processing of glare via the menace response and dazzle reflex^45^, respectively (after confirming that cranial nerve V and VII reflexes were intact), (ii) conscious proprioception via hoof placement, knuckling and hopping tests, (iii) body condition scoring, and (iv) spinal cord integrity via cutaneous trunci reflex testing. In accordance with UK Home Office regulations (IACUC equivalent) the CLN1 sheep were euthanised once those signs reached a humanely defined end point with age-matched controls euthanised in parallel with each homozygote. The humanely defined end-point was selected prior to the development of any seizures in the CLN1 sheep therefore a “terminal” end-point may occur a few of months later than the humanely defined time point.

### Ethics statement

All animal work was reviewed and approved by the Animal Welfare and Ethical Review Board (AWERB) at the Roslin Institute and conducted under the authority of the UK Home Office (equivalent of IACUC).

## Supplementary information

Supplementary figures

## Data Availability

Material will be made available if appropriate requests are received.
