# Supplementary material for: CRISPR/Cas9 mediated generation of an ovine model for infantile neuronal ceroid lipofuscinosis (CLN1 disease)
Source: Sci Rep. 2019 Jul 9;9:9891. doi: 10.1038/s41598-019-45859-9 (PMC6616324; doi:10.1038/s41598-019-45859-9)
Supplement: Supplementary file 1 — Supplementary figures [file 41598_2019_45859_MOESM1_ESM.pdf]

Supplementary Information for:

CRISPR/Cas9 mediated generation of an ovine model for infantile neuronal ceroid lipofuscinosis (CLN1 disease)

Authors: Eaton SL[1#], Proudfoot C[1#], Lillico SG[1#], Skehel P[2 3], Kline RA[1], Hamer K[5], Rzechorzek NM[4 5], Clutton E[6], Gregson R[6], King T[1], O'Neill CA[7], Cooper JD[8 9], Thompson G[2], Whitelaw CB[1§], Wishart TM[1 3§] *

1. The Roslin Institute and Royal (Dick) School of Veterinary Studies, University of Edinburgh, Edinburgh, UK
2. Centre for Discovery Brain Science, University of Edinburgh, Hugh Robson Building, Edinburgh, UK.
3. Euan MacDonald Centre for Motor Neurone Disease Research, University of Edinburgh, Edinburgh, UK.
4. Centre for Clinical Brain Sciences, University of Edinburgh, Chancellor's Building, 49 Little France Crescent, Edinburgh, UK.
5. Royal (Dick) School of Veterinary Studies, The University of Edinburgh, Edinburgh. UK
6. Wellcome Trust Critical Care Laboratory for Large Animals, Roslin Institute, Easter Bush, Edinburgh. UK
7. BioMarin Pharmaceutical Inc., San Rafael, CA, USA.
8. Los Angeles Biomedical Research Institute at Harbor-UCLA Medical Center, David Geffen School of Medicine, UCLA, Torrance, CA, USA.
9. Department of Pediatrics, Washington University School of Medicine, St Louis, MO, USA.

[# §] These authors contributed equally

* Corresponding author: T.M.Wishart@ed.ac.uk

ssODN1 atctgtcttctactgctgtaggtgtttttggactccctTAAtgcccaggagaaagctcacacatctgtgacttcatcag

ssODN2 atctgtcttctactgctgtaggtgtttttggactccctTAAtgGccaggagaaagctcacacatctgtgacttcatcag

Supplementary Figure 1: Sequence of the HDR repair template provided as an ssODN

The red letters indicate sequence changes introduced in to the WT sequence. The TAA in both ssODNs introduces a coding change, the R151* mutation described in patients. The G in ssODN2 is a silent blocking mutation required to prevent the re-cutting of the repaired allele by sgRNA 2.

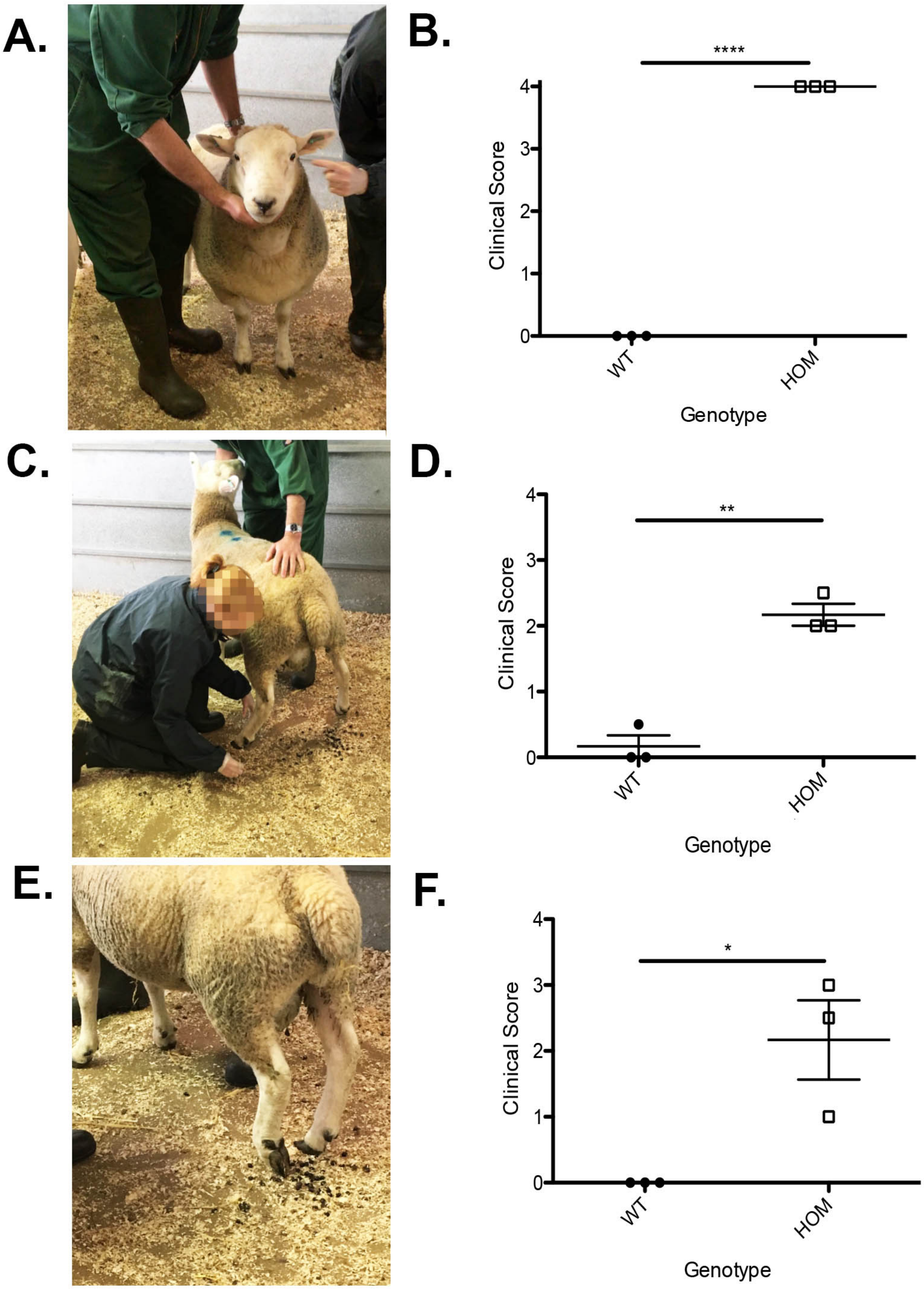

Supplementary Figure 2: PPT1 sheep show exhibit clinical signs similar to patients with CLN1 disease at humanely defined endpoint

A. Photograph demonstrating assessment of the menace response. **B.** Scatter plot demonstrates a significant loss in the menace response of the homozygote PPT1 sheep (P<0.0001). **C.** Picture demonstrates hoof placement test. **D.** Scatter plot demonstrates a significant increase in loss of hoof placement in homozygote sheep (P=0.0011). **E.** Photograph demonstrates knuckling test in rear limbs. **F.** Scatter plot demonstrates a significant loss of conscious proprioception in the homozygote sheep as indicated using the knuckling test on the hind limbs (P=0.0226). Statistical analyses utilized unpaired two-tailed Student's t-test. Error bars represent SEM.
